# The addition of the surgical robot to skin cancer management

**DOI:** 10.1308/003588413X13511609955012

**Published:** 2013-01

**Authors:** AD MacKenzie Ross, P Kumar, BJ Challacombe, P Dasgupta, JLC Geh

**Affiliations:** Guy’s and St Thomas’ NHS Foundation Trust,UK

**Keywords:** Melanoma, Squamous cell carcinoma, Merkel cell carcinoma, Robotics, Minimally invasive surgical procedures, Lymph node excision

## Abstract

We present the introduction of the surgical robot for pelvic lymphadenectomy for skin cancer through a cross-specialty collaboration. In this prospective series, we include the first report of cases undergoing robot-assisted pelvic lymph node dissection for Merkel cell carcinoma and melanoma in the recognised scientific literature.

Pelvic lymphadenectomy is advocated, sometimes prophylactically, in international guidelines for both melanoma and squamous cell carcinoma (SCC).^1–3^ Laparoscopic techniques have been applied to minimise the significant morbidity associated with open pelvic dissection.^4–6^ Laparoscopic lymph node dissection in skin cancer has been limited by concerns including the ability to perform meticulous node clearance around major vessels, port site implantation and working in previously irradiated tissue planes. The enhanced dexterity and, particularly, the magnified three-dimensional vision afforded by the da Vinci^®^ robotic system (Intuitive Surgical, Sunnyvale, CA, US) addresses several of these issues. We report our first four cases of robot-assisted pelvic lymph node dissection (RAPLND) for skin malignancy, including the first for Merkel cell carcinoma (MCC) reported worldwide, managed in a multidisciplinary setting from a tertiary referral centre using a four-arm da Vinci^®^ Si (Fig 1).

**Figure 1:**
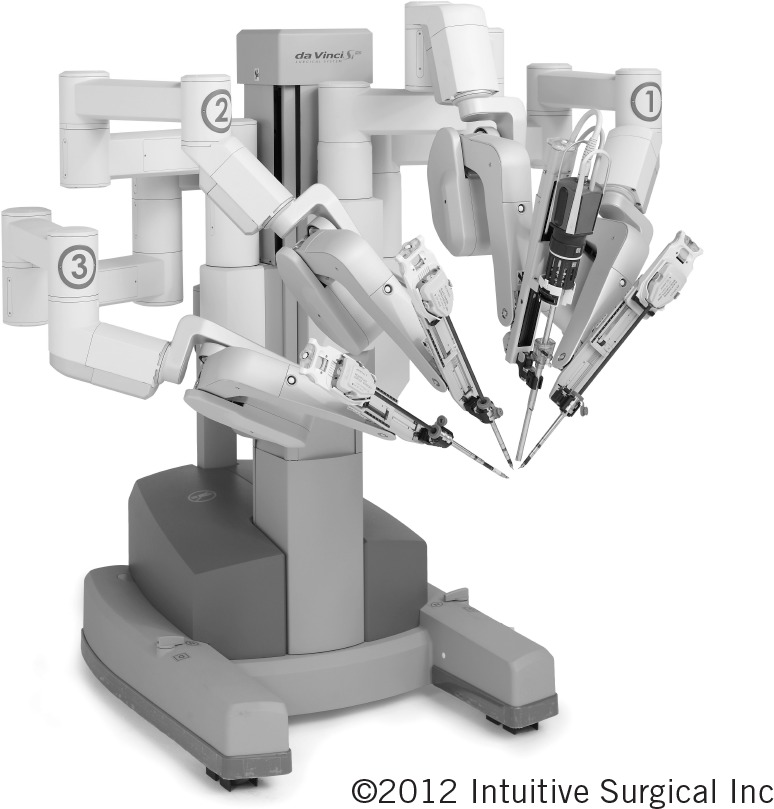
The four-arm da Vinci^®^ Si

## Methods

Data were collected prospectively for patients undergoing RAPLND for skin cancer at the Guy’s and St Thomas’ NHS Foundation Trust. These included age, indication, disease pathology, site of primary tumour, previous treatment, comorbidities, operative time, complications, length of hospital stay, the histopathologically reported number of lymph nodes and nodal disease involvement.

## Results

All operations were performed at Guy’s Hospital and all patients were female. The cases are summarised in Table 1. The first patient was offered a prophylactic pelvic clearance after her inguinal lymphadenectomy (for a stage IIIB melanoma arising from her thigh) had shown two of eight nodes with extracapsular spread. This was complicated by seroma formation, pain, cellulitis and lymphoedema. She was offered RAPLND after refusing an open approach due to the morbidity associated with the latter procedure. Ipsilateral RAPLND was carried out without complications. The patient was fit for discharge 24 hours postoperatively. The genitofemoral and obturator nerves formed the cranial and inferior borders of the dissection. All tissue medial and lateral to the external iliac vessels was cleared. All nodes retrieved were clear of metastases. She remains disease free after 15 months of follow-up.

**Table 1:** Summary of the first four reported cases to undergo robot-assisted pelvic lymph node dissection for skin cancer

	Patient 1	Patient 2	Patient 3	Patient 4
Age	69 years	86 years	43 years	74 years
Pathology	Melanoma	Melanoma	SCC	MCC
Primary site	Right thigh	Right ankle	Unknown	Right leg
Time since inguinal lymph node clearance	7 months	Not required	5 months	5 months
Co-morbidities	Inguinal dissection complicated by pain, infection, seroma and dehiscence	Folate deficiency and raised APTT, hypertension, frail	Renal transplant, myaesthenia gravis, non-melanoma skin cancers, cervical carcinoma	Diabetes mellitus, non-melanoma skin cancers
Indication	Prophylactic (2 of 8 inguinal lymph nodes with extracapsular spread)	Enlarged pelvic lymph node confirmed on PET-CT	Extensive pelvic nodal metastases on PET-CT	Enlarged pelvic lymph node confirmed on PET-CT
Operative time	90 min	90 min	150 min	90 min
Complications	Pre and postoperative lymphoedema	Ileus, constipation	Pre and postoperative lymphoedema, died 4 months later	None
Pelvic histopathology	0 of 6 nodes	1 of 5 nodes (extracapsular spread)	Mass of metastatic SCC	1 of 8 nodes (extracapsular spread)
Length of hospital stay	4 days*	17 days	8 days	2 days

SCC = squamous cell carcinoma; MCC = Merkel cell carcinoma; APTT = activated partial thromboplastin time; PET-CT = positron emission tomography - computed tomography

* Medically fit to be discharged at 24 hours but patient declined as she was the first patient to undergo robotic surgery for skin cancer

The second patient was an 86-year-old woman, the oldest we know of to have a robotic procedure, presenting with a stage IIIC melanoma (three in-transit metastases and a pelvic node detected on positron emission tomography - computed tomography [PET-CT]). Minimally invasive surgery was indicated due to multiple co-morbidities including a clotting deficiency. RAPLND and concurrent right ankle metastasis excision were carried out without immediate complications. Technical modification included a ‘no touch’ technique of the golf ball sized melanoma deposit. The postoperative course was complicated by constipation and ileus but no wound complications. The patient remains disease free, independent and self-caring at home after 12 months of follow-up.

The third patient was a 43-year-old woman with a history of myaesthenia gravis, a renal transplant, multiple cutaneous SCCs and a vulval carcinoma treated previously with radiotherapy, presenting with groin swelling. A prior inguinal lymphadenectomy had been performed for a metastatic SCC with extracapsular spread. Follow-up PET-CT detected an 8cm mass adherent to the external iliac vessels in close proximity to the extraperitoneal renal transplant. RAPLND was carried out without complications. Technical modification included proximal control of the external iliac artery and vein with vessel loops enabling dissection of the lymph node mass with clear surgical margins. Sharp dissection was used in the previously irradiated field. A single venotomy was closed with a 5/0 Prolene^®^ suture (Ethicon, Somerville, NJ, US). The patient’s total intraoperative blood loss was 50ml. She succumbed to her disease four months postoperatively.

The fourth patient was a 74-year-old diabetic woman who underwent a right inguinal lymphadenectomy for a metastatic SCC. PET-CT detected a positive pelvic lymph node. Ipsilateral RAPLND was performed without complications. One of eight nodes retrieved showed MCC, a rare cancer that shows a high preponderance to lymphatic spread. Metastases should be excised where possible and radiotherapy considered.^7^ The patient remains well 11 months postoperatively.

All cases were completed robotically. Associated morbidity was low, with no complications of wound healing. In the case of Patient 3, open surgery would have been hazardous in a previously irradiated field containing a transplanted kidney.

## Discussion

The morbidity of open pelvic lymph node dissection has made this treatment modality unpopular. However, several groups have reported a 5-year survival rate of up to 47% and a 10-year survival rate of 20% in patients in patients undergoing combined inguinal and pelvic dissection.^4,8^ Lymphatic studies demonstrate trunk and thigh sentinel node drainage to lie in the pelvis in a significant proportion of patients.^9^ RAPLND offers the potential to minimise morbidity for both prophylactic and therapeutic indications for a pelvic lymphadenectomy. We know from our prostate patients that robotic surgery offers rapid patient recovery, mobilisation and return to normal activities of daily living. Surgery can be conducted safely in the previously operated/irradiated pelvis. Modern tissue retrieval systems using disposable endoscopic bags eradicate the possibility of port site seeding.

Three out of four patients in our series deferred surgery due to concerns with pain and prolonged recovery following open surgery. Once a suitable alternative technique was made available to them, they were able to be treated as per guidelines by both a skin surgeon and an experienced urological robotic team.

The particular technical demands of the robot control reward operator experience. The collaboration between skin cancer and experienced robot urology surgeons enables us to offer RAPLND to patients for whom the potential morbidity of an open operation outweighs the likely benefit of a pelvic lymphadenectomy. The cost and multidisciplinary skills required to make this technique available will, of course, restrict its use to mainly tertiary subspecialty referral units.

## Conclusions

This prospective series of patients with skin malignancy undergoing RAPLND demonstrates the potential of the technique in minimising the morbidity of a pelvic lymphadenectomy and the importance of cross-specialty collaboration in bringing technological advances to patients. We are not advocating that this becomes a standard approach in all units. This combined approach came about due to restrictions on which surgeons can operate on skin cancer patients. Guidance from the National Institute for Health and Clinical Excellence^10^ had been adhered to in these cases and the patients benefited from the extra choice. High patient satisfaction with the rapidity of recuperation following RAPLND has encouraged our unit to develop and offer this modality to patients deemed appropriate from our specialty skin cancer multidisciplinary meetings.
